# Impact of unilateral single-mode balance training vs. combined balance and plyometric training on soccer players’ interlimb asymmetry in balance and neuromuscular performance

**DOI:** 10.3389/fspor.2026.1743152

**Published:** 2026-02-19

**Authors:** Thomas Muehlbauer, Katharina Borgmann, Sam Limpach, Dirk Krombholz, Stefan Panzer

**Affiliations:** 1 Division of Movement and Training Sciences/Biomechanics of Sport, University of Duisburg-Essen, Essen, Germany; 2 Institute of Biomechanics and Orthopaedics, German Sport University Cologne, Cologne, Germany; 3 Institute of Sport Science, Saarland University, Saarbrücken, Germany; 4 Dynamo Dresden, Dresden, Germany; 5 Department of Health and Kinesiology, Texas A&M University, College Station, TX, United States

**Keywords:** cross-education, exercise, functional reach, interlimb difference, intervention, muscle activation, proprioception, stability

## Abstract

**Background:**

It has been shown that unilateral balance training (BT) enhances balance and neuromuscular performance in the trained and untrained leg and thus has the potential to reduce side-to-side differences. However, it is unresolved how effective unilateral single-mode BT compared to unilateral combined balance and plyometric training (BT + PT) is in reducing interlimb asymmetry. Thus, we examined the impact of both training modalities on soccer players’ interlimb asymmetry in balance and neuromuscular performance.

**Methods:**

Sixty male elite youth soccer players were randomly assigned to a unilateral single-mode BT group (*n* = 20), a unilateral combined BT + PT group (*n* = 22) or an active control group (*n* = 22). All groups trained for nine weeks (2 sessions/week) with their non-dominant leg. The interventions included balance exercises for the single-mode BT group, balance and plyometric exercises for the combined BT + PT group and stretching exercises for the active control group. Training progression was achieved by manipulating sensory input (balance exercises), the number of floor contacts (plyometric exercises), or the duration of the stretching exercises. Before and after the intervention period, performance in the Y Balance Test – Lower Quarter (YBT–LQ) and the single leg drop landing (SLDL) test as well as leg muscle activity were assessed. The magnitude of interlimb differences was quantified by calculating the limb symmetry index (LSI) and compared using repeated measures analysis of variance (significance level: *p* < .05).

**Results:**

The analysis revealed significant main effects of test (pre *vs.* post) for the LSI values of the YBT–LQ (anterior reach: *η*_p_^2^ = .07; posterolateral reach: *η*_p_^2^ = .11) and activity of the gastrocnemius muscle (*η*_p_^2^ = .33) but not for the SLDL test. Further, a significant test×group interaction was detected for the LSI value of the anterior reach (*η*_p_^2^ = .10) and *post-hoc* analyses yielded a significant LSI reduction for the unilateral single-mode BT group (*d* = .79) only.

**Conclusion:**

Nine weeks of unilateral single-mode BT and combined BT + PT proved to be effective and feasible (attendance rates of ≈ 99%–100%). In particular, unilateral single-mode BT appears to be suitable for reducing the LSI value of the YBT–LQ anterior reach, which is a predictor for time loss non-contact lower limb injuries.

## 1 Introduction

There is evidence (1, 2) that a significant difference (i.e., >10%–15%) between the lower limbs in postural control is associated with a loss in athletic performance. For example, Gonzalo-Skok et al. (1) reported that interlimb asymmetry in balance performance was associated with lower vertical jump height in fifteen young elite male basketball players. In addition, there is evidence of relationships between interlimb asymmetry and both technical and physical demands in soccer. Teixeira and Teixeira (3) examined the relationship between technical soccer-related tasks (i.e., dribbling, trapping, and passing the ball) and leg asymmetry in 6- to 10-year-old soccer players (*N* = 24). They reported significantly faster movements during task execution with the dominant right foot compared to the non-dominant left foot. Atkins and colleagues (4) analysed physical demands of 92 elite youth soccer players under 13 to under 18 during a bilateral deep squat exercise. The authors observed significant differences between right and left peak ground reaction forces across all age groups, with higher values on the left side.

Further and from a health perspective, an increased injury risk has also been repeatedly reported (5, 6) when a certain threshold was exceeded with regard to side-to-side difference in postural control. More precisely, Plisky et al. (5) assessed balance performance and documented the incidence of non-contact lower limb injuries in high school basketball players. Using the Y Balance Test – Lower Quarter (YBT–LQ), the authors reported that players with an anterior reach distance difference of more than 4 cm between the left and right leg were 2.5 times more likely to sustain ankle sprains and anterior cruciate ligament tears. Therefore, interventions are needed that have the potential to reduce interlimb asymmetry in postural control, thereby reducing the risk of injury and athletic performance decrements.

In fact, several studies (7–9) investigated the influence of unilateral balance training (BT) on balance and neuromuscular performance in the trained and/or untrained leg. For instance, Rasool and George (7) conducted a study with 30 healthy recreationally active male individuals (age: 22 ± 5 years), who performed unilateral BT for four weeks (five times per week). Compared to the control group, they found significant balance improvements (i.e., YBT–LQ reach distances) for the trained leg (+20.4%) and to a lesser extent for the untrained leg (+10.1%). In another study by Oliveira et al. (8), 23 healthy recreationally active male adults (age: 28 ± 4 years) performed unilateral BT over six weeks (four sessions per week, 25 min per session). Again, the researchers reported significant enhancements in balance performance (i.e., reduced postural sway) for the trained leg (≈35%) and in a lower degree for the untrained leg (≈6%) in comparison to the control group (≈8%). Further, muscular onsets were significantly reduced after training for both the trained (≈16%) and untrained (≈14%) leg but with no significant changes in muscle activation for the control group (≈3%). Further neuromuscular adaptations as a result of BT (e.g., reduced motor evoked potential size, reduced maximal H-reflexes) have been reported in previous studies (10, 11). These findings are promising, as direct effects on the trained leg (ipsilateral) as well as transfer effects (cross-education) on the untrained leg (contralateral) were reported. However, a pre-training vs. post-training comparison concerning side-to-side differences in postural control was not conducted. Thus, it remains unclear whether unilateral BT is effective to reduce interlimb asymmetry in balance and neuromuscular performance. In addition, the training modalities applied in the present study, as well as those discussed in recent reviews (12–14), were limited to balance exercises only, although evidence (15–18) suggests that a combination of balance and plyometric training (BT + PT) is more effective than single-mode training, as adaptive resources from both training modalities are integrated. In addition, previous studies (7, 8, 19–21) exhibit methodological limitations, including the investigation of relatively small sample sizes, injured individuals or older adults, and the assessment of behavioural rather than neuromuscular outcomes.

Therefore, the aim of this study was to investigate the impact of nine weeks (2 sessions per week) unilateral single-mode BT vs. unilateral combined BT + PT when conducted in combination with regular soccer training on male elite young soccer players' interlimb asymmetry in balance and neuromuscular performance. This comparison enables statements on the effectiveness (i.e., reduced interlimb asymmetry in postural control ≈ behavioural level) as well as the underlying adaptations (i.e., reduced interlimb asymmetry in muscle activity ≈ neuromuscular level) of different training modalities (unilateral single-mode BT *vs.* unilateral combined BT + PT). The received knowledge can be used by practitioners (e.g., coaches, therapists) for the design of more effective and efficient training procedures in order to reduce interlimb asymmetry in postural control. Based on the results of the aforementioned studies, we hypothesised that both unilateral single-mode BT and unilateral combined BT + PT would lead to reductions in soccer players' interlimb asymmetry in measures of postural control and muscle activity, and these effects would be greater in the combined BT + PT due to complementary adaptations caused by the combination of balancing and explosive power activities.

## 2 Material and methods

### 2.1 Participants and sample size estimation

Using G*Power 3.1.9.7 software (22), we performed an *a priori* power analysis for a repeated measures, within-between interaction analysis of variance (ANOVA) to estimate the required sample size. Effect sizes from previous studies (8, 19, 21) investigating training effects on interlimb differences were used as a basis for the calculation. Assuming Cohen's *f* = .25, *α* = .05, 1-*β* = .80, three groups, two measurements, a correlation among repeated measures of *r* = .30, and a drop-out rate of 5% per group due to reasons not attributable to treatments, the calculation indicated that a total sample size of 60 soccer players would be sufficient to detect significant differences. Consequently, 64 male elite young soccer players were recruited from the under 13 (i.e., 11–12-year-olds), under 15 (i.e., 13–14-year-olds), and under 17 (i.e., 15–16-year-olds) teams of a soccer club (i.e., SG Dynamo Dresden e. V.) and allocated to a unilateral single-mode BT group (*n* = 20), a unilateral combined BT + PT group (*n* = 22) or an active control group (*n* = 22) (Table 1). We applied cluster randomization to distribute an equal number of players from each age category across the groups. Precisely, 6–8 players from each team were randomly assigned to one of the three groups. All players were free of any musculoskeletal dysfunction, neurological impairment, or orthopaedic pathology within the preceding three months, and they played in the highest league for their age category. Player's assent and written informed consent of the parents or legal guardians were obtained before the start of the study. The Human Ethics Committee at the University of Duisburg-Essen, Faculty of Educational Sciences approved the study protocol (approval number: TM_04.06.2020).

**Table 1:** Characteristics of the male elite young soccer players by group (*N* = 64)^a^.

Characteristic	Single-mode BT group	Combined BT + PT group	Active control group	*p*-value
Sample size (*n*)	20	22	22	–
Mean age (years)	14.0 ± 1.6	14.4 ± 1.6	14.2 ± 1.7	.759
Maturity offset (years from PHV)^b^	0.08 ± 1.46	0.41 ± 1.63	0.21 ± 1.76	.812
Body height (cm)	169.1 ± 9.7	171.7 ± 12.8	169.5 ± 12.5	.752
Body mass (kg)	57.5 ± 12.7	58.7 ± 14.4	58.8 ± 15.3	.947

Values are means ± standard deviations.

^a^ One player in the unilateral single-mode balance training group was excluded from analysis because he missed the pre-test and three players in the active control group were removed because they missed the post-test.

^b^ Maturity offset was calculated as years from peak height velocity by using the formula provided by Mirwald et al. (42). BT, unilateral balance training; BT + PT, unilateral balance and plyometric training; PHV, peak height velocity.

### 2.2 Testing procedure

**Figure 1:**
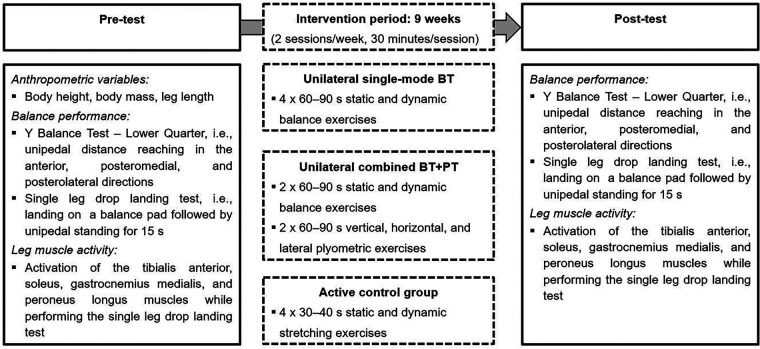
Schematic description of the study design. Unilateral practice was performed with the non-dominant leg (i.e., stance leg while kicking a ball). BT, unilateral balance training; BT + PT, unilateral balance and plyometric training.

The design of the present study is displayed in Figure 1. Both, the pre-test and post-test were conducted in the training facilities of the soccer team by the same skilled assessors (graduated sport scientists). All soccer players received standardised verbal instructions and a visual demonstration regarding the applied assessment that included anthropometric variables, balance outcomes, and electromyographic (EMG) data. Prior to each test date, all players underwent a standardised 10-minutes warm-up procedure, which consisted of balance and submaximal plyometric exercises.

### 2.3 Interventions

The three groups completed a 9-week intervention (2 sessions/week, 30 min per session) during the competitive period, instructed and supervised by the respective certified athletic coaches of the club (Table 2). The unilateral single-mode BT group performed static and dynamic exercises using balance boards, spinning tops, BOSU balls, and balance pads (23) while standing on the non-dominant leg (i.e., stance leg while kicking a ball). One set of exercise consisted of four sets of 60–90 s exercise duration alternated with 30 s rest periods. Difficulty of BT was increased by extending the duration per exercise and by manipulating the sensory input (i.e., transfer from standing on firm/stable to foam/unstable surface) (24). For the unilateral combined BT + PT group, each training session was divided into two parts. The first part consisted of the exercise description mentioned before but reduced to two sets of 60–90 s exercise duration per set of exercise and the second part included vertical, horizontal, and lateral plyometric tasks (e.g., box, hurdle, squat jumps) (25). Training progression was ensured by an incremental increase in jump (hurdle) height from 10 to 90 cm. The total number of ground contacts per session amounted to 108–162 for weeks 1–2, 90–168 for weeks 3–4, 114–166 for weeks 5–6, 108–162 for weeks 7–8, and 116–216 for week 9 and corresponds to values reported in other studies with young soccer players (26, 27). The participants of the active control group conducted passive and active stretching exercises for the upper (i.e., core, pectoral, and shoulder muscles) and lower (i.e., calf, quadriceps, hamstring, and hip muscles) body. Each set of exercise involved four repetitions of 30–40 s exercise duration alternated with 30 s rest periods. The stretching programme was intensified by increasing the duration of a single exercise and by changing from static to dynamic movement execution. Additionally, all three groups performed their usual training routine that consisted of soccer-specific training (360–420 min/week) and athletic training (90–180 min/week) and one game per weekend. More precisely, soccer-specific training sessions were conducted on match day +2, −4, −3, −2, and −1, while athletic training sessions took place on match day +2 and −3. Moreover, the players participated in daily physical education lessons from Monday to Friday from 08:00 to 10:45 a.m. For all three groups, training load (i.e., frequency, duration, volume, intensity, and progression) was monitored by recording relevant parameters (e.g., exercise duration, number of ground contacts etc.) on standardized scoring sheets and regulated through weekly phone calls between the study examiner and the certified athletic coaches.

**Table 2:** Description of the 9-week (2 sessions/week, 30 min per session) in-season intervention period by group.

Group	Weeks 1–2	Weeks 3–4	Weeks 5–6	Weeks 7–8	Week 9
Unilateral single-mode BT group	Balance exercises				
	4 × 60–90 s upright stance on a balance board (**level 2**)	4 × 60–90 s upright stance on a balance board (**level 3**)	4 × 60–90 s upright stance on a balance board (**level 4**)	4 × 60–90 s upright stance on a balance board (**level 5**)	4 × 60–90 s upright stance on a balance board (**level 6**)
	4 × 60–90 s **for-/ backward** leaning on **firm** ground	4 × 60–90 s **sideward** leaning on **firm ground**	4 × 60–90 s **for-/ backward** leaning on **foam** ground (AIREX® balance pad)	4 × 60–90 s **sideward** leaning on **foam** ground (AIREX® balance pad)	4 × 60–90 s **rotational** movements on **foam** ground (AIREX® balance pad)
	4 × 60–90 s distance reaching (AT, PM, PL) on **firm** ground	4 × 60–90 s distance reaching (AT, PM, PL) on **foam** ground (AIREX® balance pad)	4 × 60–90 s distance reaching (AT, PM, PL) on **foam** ground (ball cushion)	4 × 60–90 s distance reaching (AT, PM, PL) on **foam** ground (BOSU ball)	4 × 60–90 s distance reaching (AT, PM, PL) on **unstable** ground (spinning top)
	4 × 60–90 s **horizontal** jump-landings on **firm** ground	4 × 60–90 s **sideward** jump-landings on **firm** ground	4 × 60–90 s **vertical** jump-landings on **firm** ground	4 × 60–90 **horizontal** jump-landings on **foam** ground (AIREX® balance pad)	4 × 60–90 **vertical** jump-landings on **foam** ground (AIREX® balance pad)
Unilateral combined BT + PT group	**Balance exercises**				
	2 × 60–90 s upright stance on a balance board (**level 2**)	2 × 60–90 s upright stance on a balance board (**level 3**)	2 × 60–90 s upright stance on a balance board (**level 4**)	2 × 60–90 s upright stance on a balance board (**level 5**)	2 × 60–90 s upright stance on a balance board (**level 6**)
	2 × 60–90 s **for-/ backward** leaning on **firm** ground	2 × 60–90 s **sideward** leaning on **firm** ground	2 × 60–90 s **for-/ backward** leaning on **foam** ground (AIREX® balance pad)	2 × 60–90 s **sideward** leaning on **foam** ground (AIREX® balance pad)	2 × 60–90 s **rotational** movements on **foam** ground (AIREX® balance pad)
	2 × 60–90 s distance reaching (AT, PM, PL) on **firm** ground	2 × 60–90 s distance reaching (AT, PM, PL) on **foam** ground (AIREX® balance pad)	2 × 60–90 s distance reaching (AT, PM, PL) on **foam** ground (ball cushion)	2 × 60–90 s distance reaching (AT, PM, PL) on **foam** ground (BOSU ball)	2 × 60–90 s distance reaching (AT, PM, PL) on **unstable** ground (spinning top)
	2 × 60–90 s **horizontal** jump-landings on **firm** ground	2 × 60–90 s **sideward** jump-landings on **firm** ground	2 × 60–90 s **vertical** jump-landings on **firm** ground	2 × 60–90 **horizontal** jump-landings on **foam** ground (AIREX® balance pad)	2 × 60–90 **vertical** jump-landings on **foam** ground (AIREX® balance pad)
	**Plyometric exercises**				
	2 × 60–90 s squat jumps (height: **10 cm**; ground contacts: 12–18)	2 × 60–90 s countermovement jumps (height: **15 cm**; ground contacts: 16–24)	2 × 60–90 s standing long jumps (distance: **10 cm**; ground contacts: 30–40)	2 × 60–90 s ankle jumps (height: **10 cm**; ground contacts: 24–36)	2 × 60–90 s drop jumps (height: **25 cm**; ground contacts: 30–44)
	2 × 60–90 s box jumps (height: **10 cm**; ground contacts: 24–36)	2 × 60–90 s box jumps (height: **15 cm**; ground contacts: 24–36)	2 × 60–90 s box jumps (height: **20 cm**; ground contacts: 20–30)	2 × 60–90 s box jumps (height: **25 cm**; ground contacts: 20–30)	2 × 60–90 s box jumps (**variable** heights of 30–90 cm; ground contacts: 18–36)
	2 × 60–90 s lateral hurdle jumps (height: **10 cm**; ground contacts: 40–60)	2 × 60–90 s lateral hurdle jumps (height: **15 cm**; ground contacts: 40–60)	2 × 60–90 s lateral hurdle jumps (height: **20 cm**; ground contacts: 36–54)	2 × 60–90 s lateral hurdle jumps (height: **25 cm**; ground contacts: 36–54)	2 × 60–90 s lateral hurdle jumps (**variable** heights of 10–32 cm; ground contacts: 36–72)
	2 × 60–90 s horizontal hurdle jumps (height: **10 cm**; ground contacts: 32–48)	2 × 60–90 s horizontal hurdle jumps (height: **15 cm**; ground contacts: 32–48)	2 × 60–90 s horizontal hurdle jumps (height: **20 cm**; ground contacts: 28–42)	2 × 60–90 s horizontal hurdle jumps (height: **25 cm**; ground contacts: 28–42)	2 × 60–90 s horizontal hurdle jumps (**variable** heights of 10–32 cm; ground contacts: 32–64)
Active control group	**Stretching exercises**				
	3 ROM exercises for the pectoral muscles, 4 × 30–40 s	3 ROM exercises for the core muscles, 4 × 30–40 s	3 ROM exercises for the shoulder girdle, 4 × 30–40 s	3 ROM exercises for the pectoral muscles, 4 × 30–40 s	3 ROM exercises for the core muscles, 4 × 30–40 s
	3 ROM exercises for the calf muscles, 4 × 30–40 s	3 ROM exercises for the quadriceps muscles, 4 × 30–40 s	3 ROM exercises for the calf muscles, 4 × 30–40 s	3 ROM exercises for the quadriceps muscles, 4 × 30–40 s	3 ROM exercises for the calf muscles, 4 × 30–40 s
	3 ROM exercises for the hip muscles, 4 × 30–40 s	3 ROM exercises for the hamstring muscles, 4 × 30–40 s	3 ROM exercises for the hip muscles, 4 × 30–40 s	3 ROM exercises for the hamstring muscles, 4 × 30–40 s	3 ROM exercises for the hip muscles, 4 × 30–40 s

Bold font indicates differences in task difficulty. Participants in the single-mode BT group and the combined BT + PT group were instructed to move their arms freely and to their advantage while performing the exercises. The balance board (Wobblesmart©, Artzt GmbH, Dornburg, Germany) is equipped with a mechanically adjustable pivot to increase task difficulty from level 1 (low) to 6 (high). AT, anterior; BOSU, BOth Sides Utilized; BT, balance training; BT + PT, balance and plyometric training; PL, posterolateral; PM, posteromedial; ROM, range of motion.

### 2.4 Assessments of anthropometric variables

Body height was assessed without shoes to the nearest 0.5 cm with a stadiometer (seca 217, Basel, Switzerland) and body mass was determined in light clothing and without shoes to the nearest 100 g with an electronic scale (seca 803, Basel, Switzerland). Furthermore, the length of the left and right leg was determined by measuring the distance (in cm) from the anterior superior iliac spine to the most distal aspect of the medial malleolus with the participant lying supine (28).

### 2.5 Assessment of balance performance

To assess balance performance, soccer players performed the YBT–LQ using the Y Balance test kit (Functional Movement Systems, Chatham, USA). The test kit consists of a stance platform and three pipes that are attached to the platform. The three pipes represent the anterior (AT), posteromedial (PM), and posterolateral (PL) reach directions and are marked in 1.0 cm increments for measurement purposes. Each pipe was equipped with a moveable reach indicator. The participants' task was to stand with one leg on the platform and to reach with the other leg as far as they could in the AT, PM, and PL reach directions while maintaining balance with hands placed on hips. Each participant was instructed to perform three practice trials followed by three data-collection trials per leg. During all trials, participants were asked to gaze at a black cross that was attached to a wall (3 m away) and adjusted to individual eye level. Beginning with the AT reach direction, the task was repeated for the PM and PL reach directions. A trial was rated as invalid if the participants (a) lose their balance (i.e., stepped with the reach leg on the floor), (b) lifted the stance leg from the stance platform, (c) stepped on top of the reach indicator for support, (d) kicked the reach indicator, or (e) removed their hands from the hips (28). If an invalid trial occurred, the data was discarded, and the trial was repeated until a total of three valid trials was obtained. The best trial (i.e., maximal reach distance in cm) per leg and reach direction was used for further analyses. The YBT–LQ is a valid and reliable test to assess balance performance (28, 29).

The single leg drop landing (SLDL) test was additionally used to assess balance performance in soccer players. Precisely, the players were asked to stand with one leg on a box (height: 40 cm), drop down, and land with the opposite leg on a balance pad (Airex AG, Sins, Switzerland) that was placed on top of a force plate (Kistler, Model 9260AA, Winterthur, Switzerland). After landing, the participants' task was to stand as still as possible for 15 s with hands placed on hips. Two practice trials followed by three data-collection trials (sampling frequency: 1,000 Hz) were performed, and the mean was used for further analyses. The participants were asked to gaze at a black cross during all trials. The cross was fixed to a wall at a distance of 3 m and adjusted to the individual's eye level. A trial was discarded and recollected if participants (a) performed a jump rather than a drop landing, (b) lose their balance (i.e., touched the ground with the non-stance leg), or (c) removed their hands from the hips. Validity as well as reliability of the single leg drop landing task has been reported in previous studies (30, 31).

### 2.6 Assessment of neuromuscular performance

Leg muscle activity was assessed while performing the SLDL test from five, six, and six players of the unilateral single-mode BT group, unilateral combined BT + PT group, and active control group, respectively. This relatively small number of players per group resulted from the necessity to interrupt training as little as possible for assessments during the competitive period. Circular (diameter: 10 mm), pre-gelled, self-adhesive bipolar surface electrodes (Blue Sensor, Ambu, Balerup, Denmark) were placed over tibialis anterior, soleus, gastrocnemius medialis, and peroneus longus muscles. Electrodes were positioned (centre to centre distance: 20 mm) on the muscle bellies according to the European recommendations for surface electromyography (SENIAM) (32). Inter-electrode resistance was kept below 5 k*Ω* (measured via EMG electrode impedance tester) by shaving, slightly roughening, degreasing, and disinfecting the skin. Pulling artefacts was avoided by properly fixing the electrode cables with fishnet tubes. Following attachment, the EMG signal was examined through frequency analysis and visual verification of the raw EMG. Prior to the drop landings, all athletes performed dynamic maximum voluntary contractions by executing three squat jumps from a 90-degree bend knee position. EMG signals were amplified by a factor of 1,000 and recorded telemetrically (transmission frequency: 2.4 GHz) using a biovision® (Wehrheim, Germany) EMG signal processor connected to a stationary computer running a custom built DASYLab (Menden, Germany) worksheet with a sampling frequency of 1,000 Hz per channel. Synchronization of ground reaction force and electromyographic data was achieved by a custom-written MATLAB software version 2023a (The MathWorks, Natick, MA, USA) script, which sent a 5 V signal from an Arduino to the EMG signal processor, triggering the start of the EMG data collection simultaneously with the commencement of data collection from the force plate. After 15 s, the Arduino ceased signal transmission, and the data collection was halted in DASYLab software version 2022 (Geitmann Messtechnik GmbH & Co. KG, Menden, Germany), coinciding with the termination of data collection from the force plate.

### 2.7 Data analyses

With regard to the SLDL test, ground reaction force data were low-pass filtered (cut-off frequency: 10 Hz) with a second-order Butterworth filter using a script programmed with MATLAB software. Thereafter, time to stabilisation was calculated and corresponds to the time it takes for a person to return to a stable state following single leg drop landings (30). There is a variety of calculation methods, and we decided to compute the sequential average (SA) as this is the most reliable method for both the anteroposterior (AP) and mediolateral (ML) sway directions (31).

Regarding the YBT–LQ, the normalised [% leg length (LL)] maximal reach distance per reach direction and leg was computed by dividing the absolute maximal reach distance (cm) by LL (cm) and then multiplying by 100. In addition, the normalised (% LL) composite score was computed for each leg as the sum of the three maximal reach distances (cm) per reach direction divided by three times LL (cm) and then multiplied by 100.

Concerning leg muscle activity, the data were processed using MATLAB software. In accordance with the SENIAM guidelines (33), the EMG signals were filtered (low-pass: 500 Hz, high-pass: 10 Hz; 2nd Butterworth filter) and full-wave rectified. The EMG data were then trimmed according to the time to stabilisation, which had been calculated beforehand from the SLDL test. To this end, the players were asked to perform three maximal jumps, separated by one minute, and the best trial in terms of muscle activity was selected for normalization. To create the linear envelope of the EMG signals, the root-mean-square was calculated using a gliding-window technique with a time window of 50 ms. The EMG data were then quantified by integrating the full-wave rectified signals.

In terms of interlimb asymmetry, balance and EMG data were used to calculate the limb symmetry index (LSI) in accordance with the equation reported by Bishop and colleagues (34): LSI = (1 – non-dominant leg/dominant leg) * 100. A LSI < 10% is indicative of a regular interlimb asymmetry and a value above the cut-off value indicates irregular interlimb asymmetry (34).

### 2.8 Statistical analyses

Descriptive data are presented as group mean values ± standard deviations (*SD*). The data were analysed using the Statistical Package für Social Sciences version 27.0 (IBM Inc., Chicago, IL, USA) (35). Assumptions of normality (Shapiro–Wilk test) and homogeneity of variance/sphericity (Mauchly test) were checked and met prior performing parametric analyses. Afterwards, a one-way analysis of variance (ANOVA) was computed to analyse baseline differences between the three groups. Thereafter, a series of two-way ANOVAs were performed to determine the within-subject [× 2 (pre-test *vs.* post-test)] and between-subjects [× 3 (unilateral single-mode BT group *vs.* unilateral combined BT + PT group *vs.* active control group)] effects. If significant test×group interactions were identified, pairwise comparisons (i.e., *post-hoc* tests) with Bonferroni-adjusted alpha levels were used to localise differences. Effect sizes for ANOVAs were expressed as partial eta-squared value (*η*_p_^2^) and interpreted as small (.02 ≤ *η*_p_^2^ ≤ .12), medium (.13 ≤ *η*_p_^2^ ≤ .25), or large (*η*_p_^2^ ≥ .26) (36). Pairwise comparisons were indicated with Cohen's *d* (36) and interpreted as trivial (0 ≤ *d* ≤ .19), small (.20 ≤ *d* ≤ .49), moderate (.50 ≤ *d* ≤ .79), or large (*d* ≥ .80). The alpha level for all tests was set *a priori* at *α* = 5%.

## 3 Results

All soccer players received treatments as allocated. During the 9-week intervention period, one player in the unilateral single-mode BT group was excluded from analysis due to missing the pre-test. Another three players in the active control group were removed from analysis because they missed the post-test. Thus, 60 players completed the intervention period. Attendance rates amounted to 100%, 99%, and 99% for the unilateral single-mode BT group, the unilateral combined BT + PT group, and the active control group, respectively. During the intervention phase, a total of 20 lower-limb non-contact time-loss injuries occurred, with six, six, and eight injuries in the U13, U15, and U17 teams, respectively. Over the course of the competitive season, ten lower-limb non-contact time-loss injuries occurred in the U13 team (3.0 injuries per 1,000 match hours), 38 injuries in the U15 team (8.2 injuries per 1,000 match hours), and 36 injuries in the U17 team (10.7 injuries per 1,000 match hours). Table 3 shows the group mean ± standard deviation values and Table 4 presents the ANOVA outputs for all assessed variables. No statistically significant differences were observed in pre-test values between the three groups.

**Table 3:** Intervention-related changes in the limb symmetry index for measures of balance and neuromuscular performance by group.

Outcome	Single-mode BT group		Combined BT + PT group		Active control group	
	Pre-test	Post-test	Pre-test	Post-test	Pre-test	Post-test
YBT–LQ						
LSI: AT reach [%]	9.2 ± 8.4	4.1 ± 3.1	5.3 ± 4.3	5.1 ± 4.6	6.0 ± 5.4	5.7 ± 5.6
LSI: PM reach [%]	4.8 ± 4.9	3.4 ± 3.7	5.4 ± 5.8	4.5 ± 3.6	3.9 ± 3.2	5.1 ± 4.9
LSI: PL reach [%]	6.6 ± 6.4	3.8 ± 3.4	5.9 ± 5.4	4.9 ± 4.6	6.1 ± 5.5	4.0 ± 3.3
LSI: CS [%]	4.8 ± 4.6	3.3 ± 2.7	4.3 ± 4.0	3.5 ± 2.7	3.7 ± 2.8	3.3 ± 3.2
SLDL						
LSI: SA-AP [%]	5.5 ± 3.5	4.4 ± 2.9	4.8 ± 3.4	4.9 ± 4.5	5.4 ± 3.9	6.2 ± 8.5
LSI: SA-ML [%]	37.1 ± 33.6	28.4 ± 23.8	37.5 ± 48.7	29.0 ± 24.2	28.9 ± 21.7	24.5 ± 18.9
Muscle activity						
LSI: PEL [%]	41.5 ± 31.9	14.2 ± 11.2	45.5 ± 38.8	20.6 ± 14.3	12.6 ± 12.2	25.5 ± 20.1
LSI: TIA [%]	42.1 ± 28.1	73.1 ± 44.8	63.5 ± 49.3	37.9 ± 33.2	30.7 ± 15.8	14.6 ± 9.8
LSI: SOL [%]	86.2 ± 152.3	37.6 ± 15.8	51.1 ± 44.9	22.1 ± 19.5	33.9 ± 32.4	17.5 ± 9.9
LSI: GAS [%]	77.5 ± 79.4	27.1 ± 22.8	114.9 ± 129.1	21.8 ± 12.9	86.3 ± 100.2	19.5 ± 11.9

Data are group mean values ± standard deviations. AP, anteroposterior; AT, anterior; BT, unilateral balance training; BT + PT, unilateral balance and plyometric training; CS, composite score; GAS, m. gastrocnemius medialis; LSI, limb symmetry index; ML, mediolateral; PEL, m. peroneus longus; PL, posterolateral; PM, posteromedial; SA, sequential average; SLDL, single leg drop landing; SOL, m. soleus; TIA, m. tibialis anterior; YBT–LQ, Y Balance Test – Lower Quarter.

**Table 4:** Main and interaction effects of the repeated measures ANOVA for the limb symmetry index in terms of balance and neuromuscular performance.

Outcome	Test		Group		Test × Group	
	*F*-value	*p*-value (*η*_p_^2^)	*F*-value	*p*-value (*η*_p_^2^)	*F*-value	*p*-value (*η*_p_^2^)
YBT–LQ						
LSI: AT reach [%]	**4**.**322**	**.042** (**.07)**	.609	.547 (.07)	**3**.**153**	**.050** (**.10)**
LSI: PM reach [%]	.508	.478 (.01)	.239	.788 (.01)	2.223	.118 (.07)
LSI: PL reach [%]	**6**.**695**	**.012** (**.11)**	.089	.915 (.00)	.532	.590 (.02)
LSI: CS [%]	2.886	.095 (.05)	.195	.823 (.01)	.394	.676 (.01)
SLDL						
LSI: SA-AP [%]	.005	.943 (.00)	.513	.602 (.02)	.306	.738 (.01)
LSI: SA-ML [%]	1.759	.190 (.03)	.482	.620 (.02)	.070	.933 (.00)
Activity						
LSI: PEL [%]	3.036	.103 (.18)	.908	.426 (.12)	3.086	.078 (.31)
LSI: TIA [%]	.080	.781 (.01)	**4**.**604**	**.029** (**.40)**	1.832	.196 (.21)
LSI: SOL [%]	1.966	.183 (.12)	.984	.398 (.12)	.167	.847 (.02)
LSI: GAS [%]	**6**.**804**	**.021** (**.33)**	.174	.842 (.02)	.212	.812 (.03)

.02 ≤ *η*_p_^2^ ≤ .12 indicates small,.13 ≤ *η*_p_^2^ ≤ .25 indicates medium, and *η*_p_^2^ ≥ .26 indicates large effects. Bold values indicate statistically significant differences (*α* = 5%). AP, anteroposterior; AT, anterior; CS, composite score; GAS, m. gastrocnemius medialis; LSI, limb symmetry index; ML, mediolateral; PEL, m. peroneus longus; PL, posterolateral; PM, posteromedial; SA, sequential average; SLDL, single leg drop landing; SOL, m. soleus; TIA, m. tibialis anterior; YBT–LQ, Y Balance Test – Lower Quarter.

### 3.1 Balance performance

**Figure 2:**
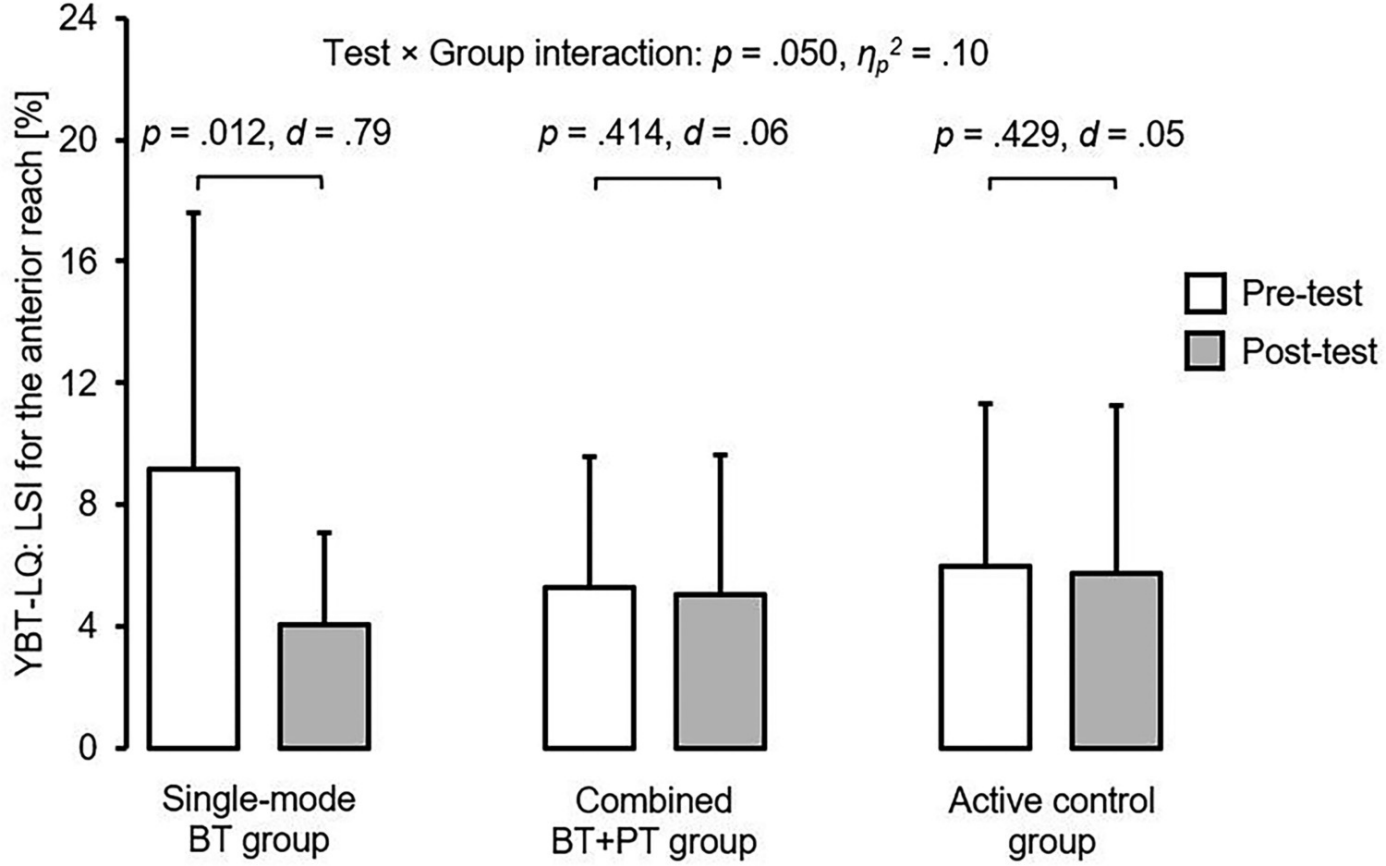
Intervention related changes in the limb symmetry index for the anterior reach direction by group. BT, unilateral balance training; BT + PT, unilateral balance and plyometric training; LSI, limb symmetry index; YBT–LQ, Y Balance Test – Lower Quarter.

For the YBT–LQ, there was a significant main effect test for the anterior (*p* = .042, *η*_p_^2^ = .07) and the posterolateral (*p* = .012, *η*_p_^2^ = .11) reach directions, indicating training-related reductions in interlimb asymmetry (irrespective of group). Further, we observed a significant test×group interaction (*p* = .050, *η*_p_^2^ = .10) for the anterior reach direction. *Post-hoc* tests revealed a significant decrease in interlimb asymmetry for the unilateral single-mode BT group (*t* = 2.471, *p* = .012, *d* = .79) but not for the unilateral combined BT + PT group (*t* = .219, *p* = .414, *d* = .06) and the active control group (*t* = .182, *p* = .429, *d* = .05) (Figure 2). The main effect of group did not reach the level of statistical significance (*p*-values ≥ .547, *η*_p_^2^-values ≤ .07). In terms of the SLDL test, there was no significant main effect of both test (*p*-values ≥ .190, *η*_p_^2^-values ≤ .03) and group (*p*-values ≥ .602, *η*_p_^2^-values = .02) as well as no significant interaction between the two factors (*p*-values ≥ .738, *η*_p_^2^-values ≤ .01).

### 3.2 Neuromuscular performance

Sub-group analyses (*n* = 17 players) revealed a significant main effect of test for the gastrocnemius medialis muscle (*p* = .021, *η*_p_^2^ = .33) and indicates reductions in interlimb asymmetry following training, independent of group. Additionally, we detected a significant main effect of group for the tibialis anterior muscle (*p* = .029, *η*_p_^2^ = .40) with lower LSI-values in the active control group compared to the other two groups, irrespective of test date. Further, there were no significant test×group interactions (*p*-values ≥ .078, *η*_p_^2^-values ≤ .031).

## 4 Discussion

The main findings can be summarised as follows: (1) Nine weeks of training proved to be feasible with high attendance rates (≈ 99%–100%) and low dropout numbers (i.e., one player in the unilateral single-mode BT group and three players in the active control group); (2) LSI values for the YBT–LQ anterior reach distance, a predictor for non-contact lower extremity injuries, were only significantly reduced following unilateral single-mode BT; (3) Irrespective of group, LSI values for the YBT–LQ posterolateral reach distance were significantly reduced; (4) Regarding the SLDL test, LSI values for the gastrocnemius muscle activation were significantly reduced (independent of group), whereas associated balance parameters did not change.

The findings of the present study indicate significant reductions in LSI values for the YBT–LQ reach distance in male elite young soccer players. This finding is in line with the literature (7, 8, 19–21) investigating the effect of unilateral BT on unipedal balance performance. For instance, Rasool and George (7) conducted unilateral BT for four weeks in male athletes (age: 22 ± 5 years) and found significant improvements (i.e., YBT–LQ reach distances) for the trained leg and to a lower extent for the untrained leg. In sum, the findings of the present and previous work (7) indicate that unilateral BT alone or in combination with explosive power exercises has the potential to significantly improve various indices (i.e., YBT–LQ reach distance) of ipsilateral and/or contralateral (i.e., cross-education that mainly results from central adaptations 9) postural control. Regarding the underlying training-related adjustments, we assume, based on studies with adults (10, 11, 37), that mechanisms of adaptation are located at the spinal and supraspinal level (38). Specifically, decreasing reflex activities (11), reduced muscle activity (11), enhanced cortico-spinal activation (10), as well as structural and functional brain changes (37, 39), were shown following BT and/or ballistic strength training.

However, in contrast to our hypothesis, significant reductions in LSI values for the anterior reach distance of the YBT–LQ were only observed in the unilateral single-mode BT group compared to the unilateral combined BT + PT group. This is contrary to the literature (15–18) and indicates that single-mode BT, but not combined BT + PT, is effective to enhance this relevant predictor of non-contact lower extremity injuries and should therefore be implemented in the regular soccer training routine. Precisely, a few studies (5, 6) reported significant associations between interlimb asymmetry for the anterior reach direction of the YBT–LQ and an increased risk of non-contact injury. For example, Plisky et al. (5) assessed YBT–LQ performance during preseason and documented time loss injuries of the lower extremities in high school basketball players. They showed that players with an anterior left/right reach distance difference greater than 4 cm were 2.5 times more likely to sustain a lower leg injury. In another study, Smith et al. (6) screened Division I athletes with the YBT–LQ during the pre-participation examination and registered the number of lower limb injuries throughout the sport season. The authors reported that athletes with an anterior left/right reach distance difference of more than 4 cm showed a 2.2 times increased risk of sustaining a lower limb injury. Why did we observe significant reductions in LSI values for the anterior reach of the YBT–LQ in the unilateral single-mode BT group but not in the unilateral combined BT + PT group, even though greater adaptations were expected for the latter due to the integration of two effective training modalities? One possible reason could be that although the same number of repetitions were performed in total, the number of repetitions per training modality was lower in the unilateral combined BT + PT group. Consequently, the resulting training volume was not enough to generate enlarged adaptations evoked by combining balancing and explosive power activities.

LSI values obtained from the SLDL test did not significantly change over the course of training. However, the LSI values for the activity of the gastrocnemius muscle were significantly reduced (independent of group). This finding is in accordance with those from previous studies (8, 40) that investigated the effects of unilateral BT on neuromuscular performance. For instance, Osborne and colleagues (40) determined whether eight weeks of BT would alter ankle muscle onset latency in patients (age: 18–45 years) with a history of lateral ankle sprain. They found significant decreases in anterior tibialis onset latency in both the trained and untrained leg. Moreover, Oliveira et al. (8) conducted unilateral BT over six weeks with adults (age: 28 ± 4 years) and reported significantly reduced muscular onsets after training in both the trained and untrained limb. Furthermore, a recent review (9) proposed cortical interconnections that transfer adaptations between limbs. In this regard, there is evidence (41) that after unilateral training, the neuronal drive from the motor cortex to the muscles (cortical voluntary activation), mediated via cross-spinal pathways, is enhanced. These findings, along with the present study, indicate that BT alone or in combination with PT is an effective training modality to enhance muscle activation.

This study has a few limitations. First, this study cannot provide any statements about adult soccer players as only young players were examined. Second, our study findings cannot be transferred to female soccer players because only male players were examined. Third, only elite soccer players were investigated, which limits the generalisation of findings to professional players. Thus, future work should study male and female soccer players of different ages and competition levels in order to extend the present findings.

## 5 Conclusions

The present study adds further insights about the effects of unilateral single-mode BT compared to unilateral combined BT + PT on male elite youth soccer players' interlimb asymmetry in balance and neuromuscular performance. Both training modalities proved to be effective and feasible with high attendance rates and low dropout numbers. In combination with regular soccer training, both training modalities resulted in significantly reduced interlimb asymmetry in balance (i.e., reach distances for the YBT–LQ) and neuromuscular (i.e., activity of the gastrocnemius muscle) performance. For the LSI value of the YBT–LQ anterior reach, the effects were greater following unilateral single-mode BT than unilateral combined BT + PT. As this measure represents a predictor for time loss non-contact lower extremity injuries, future studies should investigate whether this training modality has the potential to reduce injury risk.

## Data Availability

The raw data supporting the conclusions of this article will be made available by the authors, without undue reservation.
